# Fusexins, HAP2/GCS1 and Evolution of Gamete Fusion

**DOI:** 10.3389/fcell.2021.824024

**Published:** 2022-01-10

**Authors:** Nicolas G. Brukman, Xiaohui Li, Benjamin Podbilewicz

**Affiliations:** Department of Biology, Technion- Israel Institute of Technology, Haifa, Israel

**Keywords:** sperm, oocyte, fusexins, gamete fusion, fertilization, HAP2/GCS1, eff-1, class II viral fusion proteins

## Abstract

Gamete fusion is the climax of fertilization in all sexually reproductive organisms, from unicellular fungi to humans. Similarly to other cell-cell fusion events, gamete fusion is mediated by specialized proteins, named fusogens, that overcome the energetic barriers during this process. In recent years, HAPLESS 2/GENERATIVE CELL-SPECIFIC 1 (HAP2/GCS1) was identified as the fusogen mediating sperm-egg fusion in flowering plants and protists, being both essential and sufficient for the membrane merger in some species. The identification of HAP2/GCS1 in invertebrates, opens the possibility that a similar fusogen may be used in vertebrate fertilization. HAP2/GCS1 proteins share a similar structure with two distinct families of exoplasmic fusogens: the somatic Fusion Family (FF) proteins discovered in nematodes, and class II viral glycoproteins (e.g., rubella and dengue viruses). Altogether, these fusogens form the Fusexin superfamily. While some attributes are shared among fusexins, for example the overall structure and the possibility of assembly into trimers, some other characteristics seem to be specific, such as the presence or not of hydrophobic loops or helices at the distal tip of the protein. Intriguingly, HAP2/GCS1 or other fusexins have neither been identified in vertebrates nor in fungi, raising the question of whether these genes were lost during evolution and were replaced by other fusion machinery or a significant divergence makes their identification difficult. Here, we discuss the biology of HAP2/GCS1, its involvement in gamete fusion and the structural, mechanistic and evolutionary relationships with other fusexins.

## Introduction

The merging of the plasma membranes of two independent cells with the subsequent formation of an individual cell containing both cytoplasmic contents mixed is known as cell-cell fusion. This biological process is mediated and finely controlled by fusion proteins, termed fusogens, which are specialized proteins capable of overcoming the energetic barriers required for the fusion to occur (Chernomordik and Kozlov, 2003) and are both necessary and sufficient to mediate membrane merging (Brukman et al., 2019). In particular, the fusion between two gametes or “gamete fusion” is one of the hallmarks of meiotic sex and its ubiquitous distribution among eukaryotes suggests an ancestral origin (Ramesh et al., 2005; Speijer et al., 2015; Radzvilavicius, 2016). The first gamete fusogen identified was HAPLESS 2/GENERATIVE CELL-SPECIFIC 1 (HAP2/GCS1) that catalyzes fertilization in flowering plants, protists and probably in some invertebrates. This protein was originally found as an essential sperm factor required for male fertility in flowering plants (Johnson et al., 2004; Mori et al., 2006; von Besser et al., 2006) being later shown that it is necessary for mating in *Chlamydomonas* and *Plasmodium* (Hirai et al., 2008; Liu et al., 2008). More recently, the *Arabidopsis* HAP2/GCS1 (*At*HAP2/GCS1) was shown to be sufficient to induce fusion of mammalian cells in culture and the infection of enveloped virus to cells (Valansi et al., 2017). Strikingly, HAP2/GCS1 shares an overall three-dimensional structure with the somatic Fusion Family (FF) proteins discovered in nematodes (Mohler et al., 2002; Sapir et al., 2007; Pérez-Vargas et al., 2014) and with class II viral glycoproteins (e.g., dengue, rubella and zika viruses) (Fédry et al., 2017; Pinello et al., 2017; Valansi et al., 2017). This superfamily of fusion proteins essential for sexual reproduction and exoplasmic merger of plasma membranes was named Fusexin. Even though the mechanisms of cell-cell and virus-cell fusion are diverse and may be mediated by different families of fusion proteins (Segev et al., 2018; Vance and Lee, 2020), the fusexins represent a remarkable case of ancestral fusogens present across the tree of life. Here we review the recent research on HAP2/GCS1-mediated gamete fusion and its functional and structural relationships with other fusexins. Furthermore, we compare the regulation of fusion processes driven by fusexins to the fertilization process in mammals, where the presence of distant members of the Fusexin superfamily is uncertain.

## Structural Similarities and Differences Between Fusexins

**FIGURE 1:**
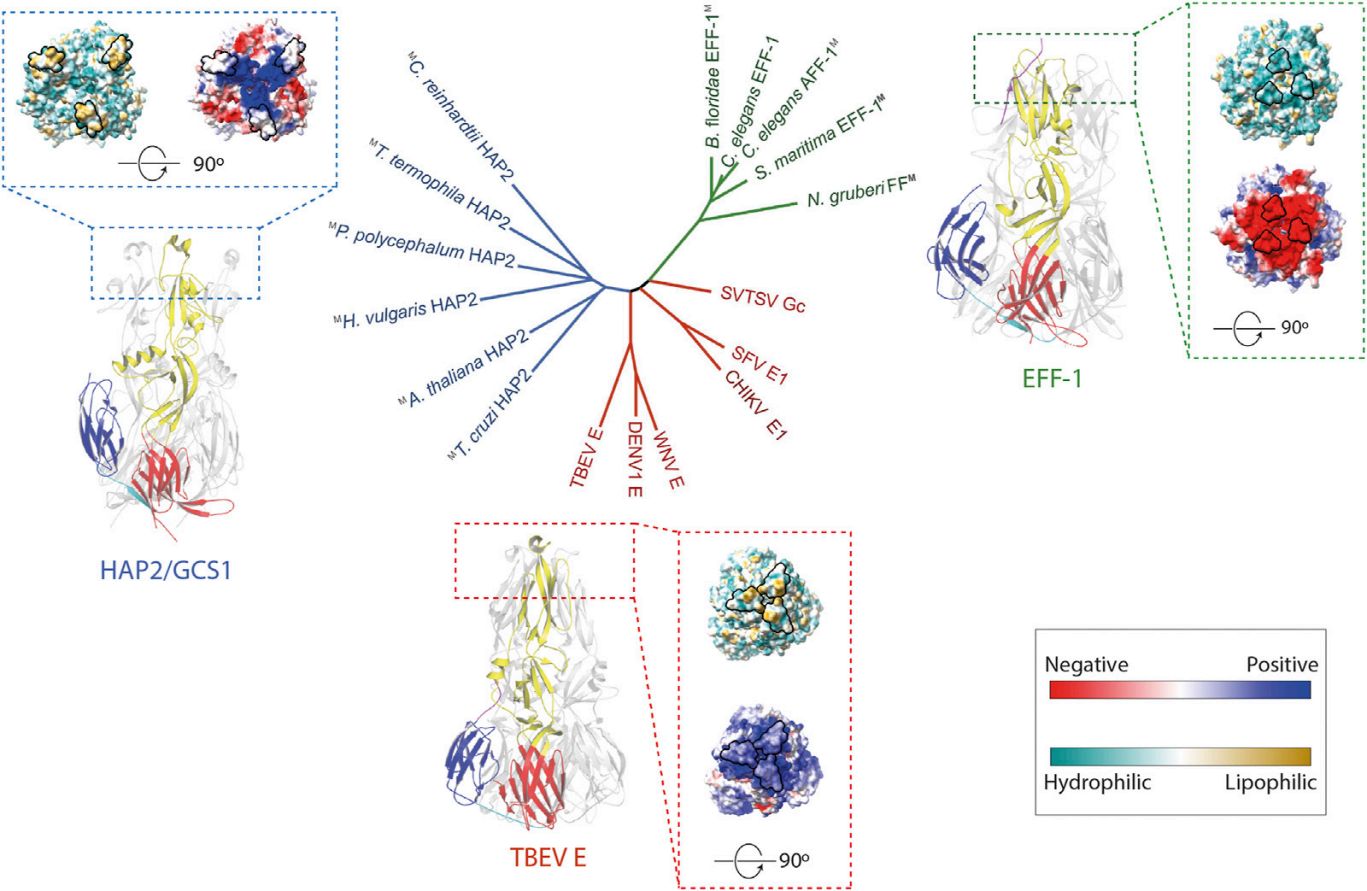
The Fusexin superfamily. Ribbon representation of the fusion protein trimers *C. elegans* EFF-1 (PDB 4OJC; Pérez-Vargas et al., 2014), TBEV E (PDB 1URZ; Bressanelli et al., 2004), and *A. thaliana* HAP2 (PDB 5OW3; Fedry et al., 2018) with domains I, II, III colored by red, yellow, blue, respectively. Surface representation of trimers viewed from the membrane was displayed. The surface is colored according to the electrostatic potential on a scale from -5 to 5 kT/e (calculated with APBS, Jurrus et al., 2018) from red (negative) to blue (positive). Solvent-excluded surfaces of trimers are colored based on molecular lipophilicity potential maps, ranging from dark cyan (hydrophilic) to dark gold (lipophilic). Proposed fusion loops at the tip of domain II are contoured in black. In the center, an unrooted tree inferred using a distance matrix extracted from (Valansi et al., 2017). Colors are HAP2/GCS1, blue; FF proteins, green; class II viral fusogens, red. M superscript represents models.

Previous studies have shown FF proteins, like *C. elegans’* EFF-1 (*Ce*EFF-1), and HAP2/GCS1 are structurally homologous to viral class II fusion proteins and display a trimeric, postfusion hairpin conformation consisting of three β-sheet-rich domains (DI, DII, DIII) (Figure 1) (Pérez-Vargas et al., 2014; Fédry et al., 2017; Pinello et al., 2017; Valansi et al., 2017). The first of the three domains consist of a β-barrel, followed by a mostly β-stranded elongated domain, and an Immunoglobulin (Ig)-like domain. They are anchored to the cell surface by transmembrane domains at the C terminus. Even though these proteins share very low sequence similarities, the surprising conservation in their architecture suggests that they share a common ancestor forming the Fusexin superfamily (Figure 1). A particular region on one end of the ectodomain has been extensively studied due to functional implications (Figure 1). In viral class II fusion proteins, fusion loops or α-helices located at the tip of domain II containing hydrophobic residues have been proposed to insert into the target membrane during conformational changes, like tick-borne encephalitis virus protein E (Bressanelli et al., 2004). In *Ce*EFF-1, the cd loop at the membrane-proximal side may play a similar structural role, however it is mainly composed of acidic residues forming an electronegative surface which is unlikely to interact with lipidic membranes (Pérez-Vargas et al., 2014). The fusion loops regions in HAP2/GCS1 orthologs are highly variable, with a single helix in *At*HAP2/GCS1, three short loops in *Trypanosoma cruzi* (*Tc*HAP2/GCS1) and three amphipathic helices in *Chlamydomonas reinhardtii* (*Cr*HAP2/GCS1) (Baquero et al., 2019). The functional relevance of the different configuration of this region is discussed in the next section.

## Mechanisms of Action of Fusexins

### One Way or Another

**FIGURE 2:**
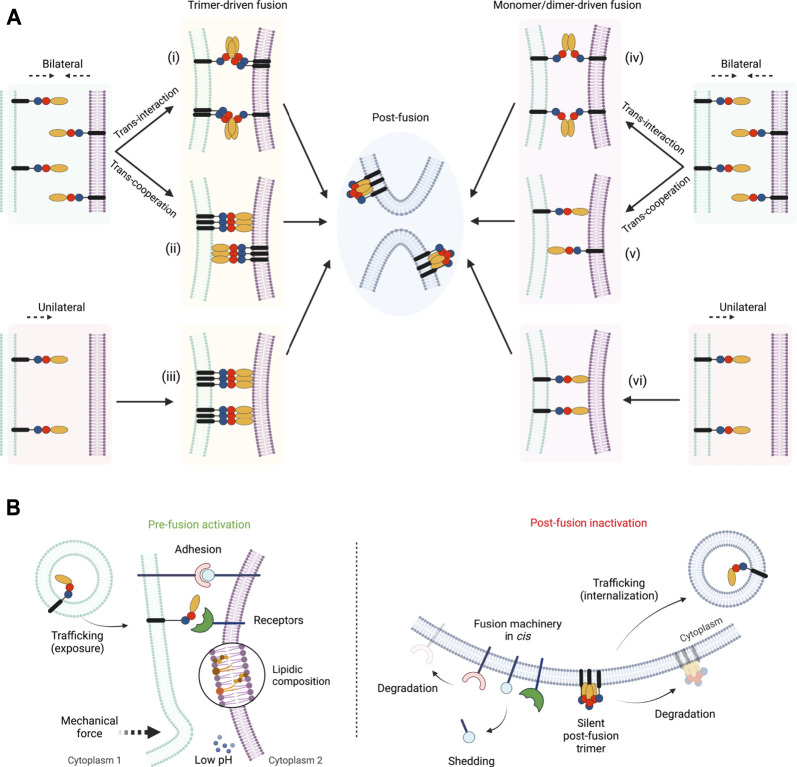
Pathways and regulations of fusexin-mediated fusion. **(A)** Different models for the mechanism of action of fusexins. Fusion can be induced from both merging membranes (“bilateral,” i, ii, iv and v) or from one of them (“unilateral,” iii and vi). Within the bilateral mechanism, fusexins may interact forming dimers or trimers leading to fusion (“trans-interaction,” reminiscent of trans-SNAREs, i and iv) or cooperate by binding to the opposed membrane (“trans-cooperation,” ii and v). Depending on the intermediate states that mediate membrane merging the mechanisms can be divided into trimer-driven fusion (i, ii and iii) or monomer/dimer-driven fusion (iv, v and vi). Independently of the mechanism, the post-fusion conformation is hairpin-shaped trimers. **(B)** Known mechanisms of pre-fusion activation (left panel) or post-fusion inactivation (right panel) of fusexins described in this review. Created with BioRender.com.

How do fusexins mediate membrane fusion? First of all, some fusogens may be required only in one of the opposing membranes, as in the case of class II viral glycoproteins (Podbilewicz, 2014), while others must be present on both sides, like the EFF-1 and AFF-1 somatic fusogens (Podbilewicz et al., 2006; Avinoam et al., 2011). These two possible mechanisms are termed unilateral and bilateral, respectively (Figure 2). However, in the case of HAP2/GCS1 the evidence is controversial. On one hand, the deletion of the *Athap/gcs1* gene induces male-specific sterility (Johnson et al., 2004; von Besser et al., 2006), even though some levels of expression were detected in ovules (Borges et al., 2008), suggesting a unilateral mechanism of action. In contrast, when studied in heterologous systems, *At*HAP2/GCS1 could mediate exoplasmic fusion only bilaterally (Valansi et al., 2017). Similarly to flowering plants, genetic studies in *Plasmodium* and *Chlamyodomonas*, showed that HAP2/GSC1 is absolutely required in only one of the fusing gametes (Hirai et al., 2008; Liu et al., 2008). By contrast, in the ciliated protozoan *Tetrahymena thermophila*, all seven mating types express HAP2/GCS1 and its absence in only 1 cell of the mating pair disrupts fusion (Cole et al., 2014; Pinello et al., 2017). The slime mold *Dictyostelium* represents a particular case where two homologs of HAP2/GCS1 were identified: HgrB, which is expressed in the three sex types, and HgrA, expressed only in types I and II (Okamoto et al., 2016). Genetic studies suggest that both HgrA and HgrB are essential in both membranes in crosses between types I and II, but only in one membrane when crossed with the third gamete type (Okamoto et al., 2016; Bloomfield, 2019).

Regardless of the species, structural and biochemical studies show that the hydrophobic tip of domain II of HAP2/GCS1 (Figure 1) is critical for interactions with liposomes (Pinello et al., 2017; Fédry et al., 2018; Feng et al., 2018; Baquero et al., 2019). This HAP2/GCS1-membrane interaction is consistent with a unilateral pathway (Figures 2Aiii,vi) but also with a bilateral mechanism with trans-cooperation (Figures 2Aii,v), or even a hybrid system. More recently, this hydrophobic region was shown to be relevant for oligomerization in *Chlamydomonas* (Zhang et al., 2021). It was shown that the formation of a small population of SDS-resistant trimers in this species was observed even in the absence of fusion, however, the temporal and spatial resolution of these experiments is not sufficient to confirm that these trimers are responsible for membrane merging.

### Which Comes First, the Fusion or the Trimer?

As mentioned before, all fusexins studied thus far can form trimers. The most studied group within the Fusexin superfamily are the viral class II fusion proteins. In mature virions of alphaviruses and flaviviruses, these proteins exist as heterodimers/trimers or homodimers, respectively, in some cases lying parallel to the membrane and forming an icosahedral lattice on its surface (Zhang et al., 2003; Voss et al., 2010). Upon internalization into the target cells, the low pH within endosomal compartments triggers the activity of class II fusogens leading to infection (White et al., 2008). It is commonly accepted that class II viral fusogens, after a transient monomeric state, trimerize before fusion and that the zippering of these trimers is the driving force for membrane merger (Baquero et al., 2013). In the post-fusion state, these proteins can be found in the already previously mentioned folded trimers of hairpins, however, the existence of the hypothetical extended trimer has not been experimentally confirmed. Therefore, the possibility that monomers rearrange into hairpins to induce fusion prior to their trimerization cannot be excluded (Figures 2Aiv,v,vi).

Even though *Ce*EFF-1 is suggested to be monomeric on cell-derived membrane vesicles (Zeev-Ben-Mordehai et al., 2014), its ectodomain purified and crystallized as a post-fusion trimer (Pérez-Vargas et al., 2014). The monomeric (but not trimeric) soluble protein was able to inhibit cell-cell fusion *in vitro* (Pérez-Vargas et al., 2014) and soluble EFF-1 trimers stimulate the fusion of insect cells ectopically expressing EFF-1 (Podbilewicz et al., 2006). Similarly to viral fusexins, EFF-1-mediated fusion can be blocked by adding soluble domain III (Pérez-Vargas et al., 2014). Based on the presence of an electronegative instead of a hydrophobic patch on the tip of the domain II and the bilateral requirement of EFF-1, it was proposed that trimers/dimers are formed in *trans* and that a bidirectional zippering occurs from both fusing membranes (Podbilewicz, 2014, Figure 2Ai,iv). However, a trans-cooperation mechanism where cis-trimers are required from both sides is still a possible model (Figure 2Aii). In the latter scenario, the binding to the opposite membrane might be indirect, distinctly to Class II viral fusexins that involve direct hydrophobic interactions with the membranes.

Whether fusion is mediated by monomers, dimers, trimers or even higher oligomeric states is not completely elucidated and different fusexins may utilize different strategies that should not be excluded (Figure 2A).

## Fine-Tuning of Gamete Fusion

Fusion must be a tightly-regulated process. Excessive gamete fusion can lead to non-specific fertilization or to polygamy that may produce unviable polyploid zygotes. Also, the fusion of gametes to their same type or to somatic cells must be avoided. In this sense, even after cell fate determination and gamete encounter, many regulatory mechanisms are established to regulate the activity of gamete fusogens.

### Attach and Fuse

Specific adhesion of the gametes seems to be a robust way of regulating fusion as fusogens require the membranes to be in close proximity (Hernández and Podbilewicz, 2017). The concept of “fertilization synapse” was introduced to describe the complex arrangement of the membranes to enable fusion (Krauchunas et al., 2016). In flowering plants, the sperm-specific protein GEX2 (Gamete EXpressed 2) contains Ig-like domains and it was shown to be essential for sperm attachment and the subsequent fertilization (Mori et al., 2014). The known *Chlamydomonas* factor for gamete adhesion is FUS1 (Ferris et al., 1996; Misamore et al., 2003), which also contains extracellular Ig-like domains that form a structure similar to the one of GEX2 (Pinello et al., 2021). In contrast to the plant counterpart, FUS1 is expressed in the plus mating type, the opposite membrane to HAP2/GCS1. However, no direct interaction between them was reported. Recently, MAR1 (Minus Adhesion Receptor 1) has been suggested to be the molecular partner of FUS1 in the minus gamete and a key regulator of HAP2/GCS1 localization to the fusion site (Pinello et al., 2021, Figure 2B). The only attachment couple known in mammals is the sperm-borne Izumo1 and the oocyte-specific Juno (Izumo Receptor). While the former is a type I transmembrane Ig-like protein, the latter is bound to the egg plasma membrane by a GPI anchor. The interaction between these two proteins leads to tight gamete binding that is species specific and is essential for fusion to occur (Inoue et al., 2005; Bianchi et al., 2014; Bianchi and Wright, 2015). This molecular interaction appears to be an evolutionary novelty as Juno is present only in mammals, however, Izumo1 has a broader distribution involving many lineages of vertebrates (Grayson, 2015). Interestingly, an Izumo-like gene involved in late stages of fertilization was described also in *C. elegans* (Nishimura et al., 2015; Takayama et al., 2021).

### The Correct Time and Place

The localization of the fusogens plays a crucial role to regulate fusion. For example, in *C. elegan*s the dynamic internalization of EFF-1 into intracellular early endosomes regulates its function (Smurova and Podbilewicz, 2017). Similarly, *At*HAP2/GCS1 localization to the plasma membrane increases upon interaction with EC1 (Egg Cell 1), a small cysteine-rich protein secreted by the female gamete, which is necessary for sperm cell plasma membrane to gain fusion competence (Sprunck et al., 2012). In mammals, the egg tetraspanin CD9 is proposed to be responsible for membrane organization and correct localization of adhesion- and fusion-related proteins, such as Juno (Inoue et al., 2020; Umeda et al., 2020). In the mammalian sperm, Izumo1 localizes into the acrosome, giant intracellular vesicle in the head. Only after undergoing capacitation, a maturation process in the female tract, the acrosomal content is released and Izumo1 relocalizes to the equatorial region of the plasma membrane where tight binding to the oocyte plasma membrane occurs (Satouh et al., 2012). The fusogens involved in mammalian gamete fusion may follow similar localization behavior to Juno and/or Izumo1.

### More Than Just Lipidic Bilayers

Another factor affecting the activity of fusion is the lipidic composition of both membranes. For example, cholesterol in the target membrane promotes the fusion mediated by some viral class II fusexins (Umashankar et al., 2008; Osuna-Ramos et al., 2018; Pattnaik and Chakraborty, 2021). On the other hand, *At*HAP2/GCS1 was reported to bind better to liposomes containing a lipid that mimics the phospholipid phosphatidylserine (Fedry et al., 2018). Phosphatidylserine is a known mediator of many fusion events (Whitlock and Chernomordik, 2021), including EFF-1-mediated neuronal repair of axons in *C. elegans* (Neumann et al., 2015) and mammalian fertilization (Rival et al., 2019). Albeit lipids may affect the activity of fusion proteins, the composition of the membrane by itself is known to determine the curvature of the bilayer and, therefore, influence directly the progression of hemifusion and pore opening (reviewed in Chernomordik and Kozlov, 2008).

### Mechanical Forces at Play

An additional element that could contribute to the cell-cell fusion process is the membrane tension by mechanical forces. For instance, it is proposed that during muscle formation in *Drosophila melanogaster* and in *Schizosaccharomyces pombe* mating, actin-rich protrusions from 1 cell are resisted by actomyosin contractions in the other, producing membrane stress that leads to fusion (Kim and Chen, 2019; Muriel et al., 2021). In this sense, actin polymerization was shown to improve EFF-1-driven fusion of insect cells in culture (Shilagardi et al., 2013). Furthermore, EFF-1 and F-actin colocalize during seam cell daughter-hyp7 cell fusion in the *C. elegans* larvae, however, this interaction seems to be important for the correct localization of the fusogen rather than a mechanical induction of fusion (Yang et al., 2017). The surface of mammalian oocytes is structurally complex and its cortex is enriched with F-actin (Longo, 1985, 1987). Nonetheless, the relevance of this actin network specifically for mammalian gamete fusion is unclear since contradicting results were reported [reviewed in (Sun and Schatten, 2006)]. It is possible that it is involved in membrane merging or in the later incorporation of the sperm. In addition, the actin cytoskeleton might control the position of the sperm during fusion, distancing it from the maternal chromosomes avoiding the elimination of paternal chromosomes during the formation of the second polar body (Mori et al., 2021). Cortical actin dynamics are also required for the correct trafficking of the cortical granules (Connors et al., 1998; Vogt et al., 2019), specialized vesicles of the oocyte that contribute to the block of polyspermy after fertilization.

Mechanical stress on the membranes can also be generated by the pushing and pulling movements of the sperm after attachment. Even though it was previously thought that motility was dispensable for mammalian gamete fusion (Yanagimachi, 1988), recent studies have suggested that a specific beating mode of the flagellum is required for this process (Ravaux et al., 2016) supporting older studies using human gametes (Wolf et al., 1995). It is also possible that these kinetic perturbations are only required for the accumulation of CD9 to the fusion site (Chalbi et al., 2014).

### Switching the Fusexins off

After fusion occurs, a rapid functional silencing of the fusogens is often required. First of all, if the fusion proteins require trans-interactions to be activated (with receptors or to other fusogens), the post-fusion configuration by itself serves as a mechanism of preventing them since all the proteins end up in the same membrane. Alternatively, the fusogens might be removed from the membrane by internalization, as is reported for *Ce*EFF-1 (Smurova and Podbilewicz, 2016), or simply by degradation, such as *Cr*HAP2/GCS1 (Liu et al., 2010). Certainly, the adhesion molecules can suffer these changes turning the membrane into fusion incompetent. For example, *Chlamydomonas* FUS1 is also degraded after fertilization (Liu et al., 2010) and the mammalian Juno is shed in extracellular vesicles from the surface of the oocyte after fusion (Bianchi et al., 2014). Other biochemical changes in the gametes triggered by fertilization may also affect the activity of the fusogens and block further fusion events. Fast changes in electric potential occur in the plasma membrane of fertilized eggs of some organisms with external fertilization, like sea urchins and frogs, which prevents polyspermy (Jaffe, 1976; Cross and Elinson, 1980). More recently, the release of inorganic zinc from mammalian fertilized oocytes was described as “zinc sparks” which can induce changes in the sperm or in the membrane of the oocyte itself (Kim et al., 2011; Duncan et al., 2016). These changes may induce conformational changes in the fusogens, in the same way that low pH induces structural rearrangements in the viral fusexins, but into an inactivating form. Finally, there is biochemical evidence for EFF-1 and viral Class II proteins that trimerization is irreversible (Bressanelli et al., 2004; Modis et al., 2004; Liao and Kielian, 2005; Nayak et al., 2009; Pérez-Vargas et al., 2014), which would mean that whenever the post-fusion conformation is reached the protein remains locked. This represents a regulatory mechanism that prevents additional fusion events from occurring. Likely this irreversibility of the trimerization is also true for HAP2/GCS1, however, these studies are still missing.

## Perspectives

Even though the overall fusexin architecture is strikingly conserved, their distribution among extremely distant organisms possibly has arisen into a diversity of mechanisms of action that might be similar or not (Figure 2). The differences in sequence, local structural features, molecular environments and interactions highlight the necessity of studying the individual biology of fusexins while avoiding overgeneralization.

On the other hand, mammals and other HAP2/GCS1-lacking organisms present an ongoing mystery. While several genes were found to be required for gamete fusion to occur (Brukman et al., 2019; Bianchi and Wright, 2020), the identity of the proteins that are both essential and sufficient for membrane merging is still unknown in fungi and vertebrates. New artificial intelligence-based tools that predict the folding of proteins (Jumper et al., 2021) may be the key to find other families of fusogens that may have replaced HAP2/GCS1, or else uncover those proteins that diverged significantly during evolution but kept the essential structure of fusexins or other families of fusogens that may have replaced HAP2/GCS1.
